# Subthreshold Diode Micropulse Laser Combined with Intravitreal Therapy for Macular Edema—A Systematized Review and Critical Approach

**DOI:** 10.3390/jcm10071394

**Published:** 2021-03-31

**Authors:** Maciej Gawęcki

**Affiliations:** Dobry Wzrok Ophthalmological Clinic, Kliniczna 1B/2, 80-402 Gdańsk, Poland; gawecki@use.pl; Tel.: +48-501-788-654

**Keywords:** combined treatment, subthreshold diode micropulse, anti-VEGF treatment, diabetic macular edema, retinal vein occlusion

## Abstract

Objective: intravitreal therapy for macular edema (ME) is a common clinical approach to treating most retinal vascular diseases; however, it generates high costs and requires multiple follow-up visits. Combining intravitreal anti–vascular endothelial growth factor (VEGF) or steroid therapy with subthreshold diode micropulse laser (SDM) application could potentially reduce the burden of numerous intravitreal injections. This review sought to explore whether this combination treatment is effective in the course of ME secondary to retinal vascular disease, and in particular, determine whether it is comparable or superior to intravitreal therapy alone. Materials and methods: the following terms and Boolean operators were used to search the PubMed literature database: subthreshold micropulse laser, subthreshold diode micropulse OR micropulse laser treatment AND anti-VEGF, anti-VEGF treatment, intravitreal steroids, OR combined therapy.This analysis included all studies discussing the combination of SDM and intravitreal anti-VEGF or steroid treatment. Results: the search revealed nine studies that met the inclusion criteria, including five comparing combined treatment and anti-VEGF treatment alone, four covering diabetic ME, and one covering ME secondary to branch retinal vein occlusion. All of these five studies suggested that combination therapy results in fewer intravitreal injections than anti-VEGF monotherapy with non-inferior functional and morphological outcomes. The remaining four studies report functional and morphological improvements after combined treatment; however, SDM alone was never superior to intravitreal-alone or combined treatment. There were substantial differences in treatment protocols and inclusion criteria between the studies. Conclusions: the available material was too scarce to provide a reliable assessment of the effects of combined therapy and its relation to intravitreal monotherapy in the treatment of ME secondary to retinal vascular disease. One assumption of note is that it is possible that SDM plus anti-VEGF might require fewer intravitreal injections than anti-VEGF monotherapy with equally good functional and morphological results. However, further randomized research is required to confirm this thesis.

## 1. Introduction

Subthreshold diode micropulse laser (SDM) therapy has been used extensively to treat retinal disorders in recent years [1,2]. The efficacy of SDM in the treatment of central serous chorioretinopathy (CSCR) has been proven in numerous studies and accepted as a routine form of treatment by many ophthalmologists in the context of this specific disease [3,4,5].

However, in other retinal disorders, especially vascular ones, current recommendations emphasize the application of intravitreal therapies. In this context, the use of SDM in these diseases remains an area to be explored. Functional improvements after SDM alone in the treatment of macular edema (ME) or diabetic ME (DME) secondary to retinal vein occlusion (RVO), can generally be described as moderate and not superior to gains achieved after intravitreal therapies [6]. On the other hand, real-world studies suggest that the actual visual gains achieved after intravitreal therapy are usually smaller than those reported in the randomized clinical trials that were the basis for the drug’s approval [7,8]. Additionally, the dense schedule of intravitreal therapy places a substantial burden on the patients, contrary to when undergoing laser treatment, which is performed less frequently. This fact was proved by reviewing five years of results of the Protocol S study by the Diabetic Retinopathy Clinical Research Network, which compared the efficacy of pan-retinal photocoagulation versus intravitreal ranibizumab for proliferative diabetic retinopathy [9]. As much as one-third of patients did not complete the trial, which often resulted in the serious progression of diabetic retinopathy. However, deterioration was much more frequent in noncompliant patients from the ranibizumab group than in those from the laser group. In light of this knowledge, the question of SDM application in retinal vascular diseases could be asked in a different way: is SDM capable of reducing the number of necessary intravitreal injections needed to maintain vision? The goal of this review was to analyze the effects of the combination of SDM and intravitreal injections in DME and ME secondary to RVO based on the available literature. In particular, the present review seeks to find premises in which to use SDM as a supportive therapy that would reduce the number of necessary intravitreal injections.

## 2. Materials and Methods

The following terms and Boolean operators together were used to search the PubMed literature database: subthreshold micropulse laser, subthreshold diode micropulse OR micropulse laser treatment AND anti-VEGF, anti-VEGF treatment, intravitreal steroids, OR combined therapy. The present analysis included all available studies that involved the combination of SDM and intravitreal anti-vascular endothelial growth factor (VEGF) or steroid treatment within the years: 2000–2021 in the PubMed database. Both SDM and anti-VEGF treatment were not available before 2000.

## 3. Results

The search revealed nine studies altogether that involved combined SDM with intravitreal treatment in ME, with the oldest one indexed in 2008. Five of these compared the results of combination treatment to those of intravitreal therapy alone. A description of these trials is presented in Table 1.

The studies compared in Table 1 consist of four studies covering DME [10,11,12,13] and one study concerning ME secondary to branch retinal vein occlusion (BRVO) [14]. Among those studies, there were two randomized clinical trials on DME by Khattab et al. [12] and Kanar et al. [13], respectively. The results of combined anti-VEGF plus SDM treatment were compared with the outcomes of anti-VEGF. These five studies reported similar best-corrected visual acuity (VA) (BCVA) and retinal morphology improvements in both groups, with significantly fewer injections required in the combined therapy cohort. Moreover, in all of these studies, SDM was performed after the loading phase of the intravitreal injection; however, the number of loading injections varied across the studies. Subsequent treatment with anti-VEGF medications was conducted in a pro re nata fashion.

Each of the remaining four studies had a unique design and they did not include intravitreal therapy alone as a reference. Nevertheless, they were analyzed because they documented the results of combined therapy. Two trials compared the outcome of combination treatment versus SDM alone [15,16], and two studies presented the effects of combination treatment in specific cases of ME [17,18]. A description of these four studies is provided in Table 2.

The triamcinolone study in ME secondary to BRVO clearly favored combined intravitreal triamcinolone (IVT) + SDM therapy over SDM alone [15]. Those patients who were subjected to combined treatment achieved better functional results than those who received SDM monotherapy. In the DME study of similar design, both the combined therapy and SDM-alone protocols proved equally effective in maintaining initial BCVA and improving retinal morphology; however, it should be remembered that baseline retinal thickness was significantly less in the SDM-alone group [16].

In the study by Elhamid et al., SDM was performed one month after intravitreal Ozurdex injection (Allergan, Dublin, Ireland) in patients with DME resistant to anti-VEGF therapy [17]. Although the results suggested the occurrence of significant morphological and functional improvements, the trial did not include a control group, so it was not possible to assess how the addition of SDM to intravitreal dexamethasone affected the final outcome. A study by Inagaki et al., which considered SDM and anti-VEGF therapy in DME is a case series, [18] observed a moderate BCVA improvement (by 0.11 logMAR), with a relatively low number of injections required to achieve this effect during one year of follow-up (mean: 3.6 ± 2.1 injections).

## 4. Discussion

Literature material for the analysis of the efficacy of the combination of SDM and intravitreal treatment in DME and RVO is scarce. Following a search of PubMed, only five eligible comparative studies were identified, including two randomized trials. Some collective findings from these studies can be reported and analyzed, although caution must be maintained. Generally, patients subjected to combined therapy required fewer injections, especially when this number was compared with the number of anti-VEGF treatments in the monotherapy population. If this outcome is confirmed in larger studies, SDM could be adopted in clinical practice to significantly reduce the burden of the treatment of retinal vascular diseases both financially and with respect to the patient’s comfort.

From the available material, it was determined that combined treatment was not inferior to anti-VEGF therapy alone when considering improvements in BCVA and retinal morphology. However, SDM was usually performed in cases of minor and moderate retinal edema or following the resolution of edema after a loading dose of the intravitreal injection was delivered. This is consistent with the results of other research correlating SDM efficacy with the amount of baseline ME, often suggesting a central retinal thickness of 400 μm as the threshold [19,20,21]. This fact implicates a strict rationale is necessary during combined SDM and anti-VEGF treatment in that the adjunct of SDM is only sensible in cases with less severe retinal edema or following a reduction in edema prompted by initial anti-VEGF therapy.

Unfortunately, the analysis of the material does not offer us a precise answer regarding what should be the treatment schedule for the combined therapy. Both the number of loading-phase injections and the moment of SDM application varied among the studies. Further research needs to address the following questions that remain: what is the optimal number of injections required during the loading phase of intravitreal therapy, what is the best time point of SDM application (e.g., complete resolution of ME, reduction below 400 μm, or reference to BCVA), and what is the ideal the retreatment schedule for either anti-VEGF or SDM? Some form of an algorithm for combined treatment in DME has already been proposed, yet it is not backed by published research [22]. SDM or anti-VEGF was suggested as the first-line therapy for DME of less than 250 μm. For larger cases of edema, an initial loading phase of two to three anti-VEGF injections followed by three injections in the context of a good response is recommended. Thereafter, switching to SDM is suggested. However, if the response is poor after two or three initial injections, a switch to SDM earlier on is indicated. Luttrull et al. does not use retinal thickness as a signal for deciding how to treat DME; if the VA is 20/50 or worse, an initial anti-VEGF injection is given and injections are continued until the VA is 20/40 or better, at which time panmacular SDM is initiated (there is no loading dose custom), while, if the VA is 20/40 or better, SDM is performed alone [23].

This review also discusses a number of non-comparative studies that do not directly refer the combined treatment to intravitreal therapy alone (Table 2). As the literature on the subject was really limited, the author attempted to evaluate each study that reported effects of combination treatment that included SDM. Two studies presented in Table 2 provide some perspective on the position of combination therapy, including SDM versus SDM alone [15,16]. It seems that SDM works well alone in mild to moderate DME; however, there is a tendency for better morphological results to be obtained with the involvement of intravitreal medication [16]. In BRVO, an additive strong anti-inflammatory effect of intravitreal steroids provided significant improvements that were clearly superior to SDM only [15]. The remaining two studies reported an effect of SDM added to either intravitreal steroid or anti-VEGF therapy in the treatment of DME [17,18]. The lack of a control groups in these reports makes their interpretation rather risky and, despite favorable morphological and functional outcomes, the benefit of adding SDM to the treatment regimen is impossible to evaluate. Moreover, it must be emphasized that intravitreal steroid therapy in the treatment of DME and ME secondary to BRVO in most cases remains the second line of therapy, as does its combination with SDM.

The author realizes that the scarceness of literature on combined treatment including both SDM and intravitreal therapy for ME does not allow for a systematic review to be performed nor for the presentation of concrete conclusions. However, in the author’s opinion, this limitation only means that this form of treatment should be looked at more carefully. The common use of intravitreal injections—anti-VEGF in particular—has pushed aside other forms of treatment, some of which are potentially effective. SDM is rarely given attention by members of industry, who support multicenter clinical trials. Thus, designing and carrying out a large SDM investigation including numerous cases is not easy and requires a lot of perseverance. Reviews such as this one will hopefully stimulate researchers to pursue the subject further.

## 5. Conclusions

An analysis of the available research on combined SDM and anti-VEGF/intravitreal treatment in ME does not provide an unequivocal answer at this time regarding the efficacy and benefits of this clinical approach. Existing published results suggest that combining SDM and anti-VEGF in the treatment of cases of limited retinal edema would reduce the number of intravitreal injections required, with functional and morphological outcomes that are non-inferior to those of anti-VEGF monotherapy. Larger, randomized clinical trials are needed to confirm this thesis and provide a rational treatment algorithm.

## Figures and Tables

**Table 1:** Studies that compared combined SDM and anti-VEGF/intravitreal steroid therapy and intravitreal treatment alone in the management of retinal diseases.

Author/Year of Publication	Material	Study Design	Results
**DME**			
Thinda et al. 2014 [10]	anti-VEGF + SDM (*n* = 10 eyes); anti-VEGF (*n* = 10 eyes)	Retrospective; evaluation of the number of injections and improvements in BCVA and CRT; follow-up of six to 18 months with a median of 12 months	Mean number of injections per month: 0.27 in the combined group and 0.67 in anti-VEGF group (difference was statistically significant); significant improvements in BCVA and final CRT similar in both groups.
Moisseiev et al. 2018 [11]	IVR + SDM (*n* = 19 eyes); IVR (*n* = 19 eyes)	Retrospective; comparison of BCVA and number of injections in both groups at 12 months and at the end of the follow-up; most patients in the SDM group had CRT < 400 µm; no more than three IVRs before SDM application.	Significant BCVA improvement similar in both groups; number of required injections was significantly fewer in the combined group than in the monotherapy group: 1.7 ± 2.3 vs. 5.6 ± 2.1 at 12 months and 2.6 ± 3.3 vs. 9.3 ± 5.1 at the end of follow-up.
Khattab et al. 2019 [12]	DME IVA (*n* = 27 eyes); SDM + IVA (*n* = 27 eyes)	Prospective, randomized; impact of adjuvant SDM therapy as compared with aflibercept treatment alone on the number of injections; evaluation of the number of injections, BCVA, and CS at 18 months; SDM applied within one week after the loading phase of injections.	Number of injections in the aflibercept group was 7.3 vs. 4.1 in the combined group (difference significant); BCVA improved significantly by a similar amount in both groups; CS improved significantly in both groups by a similar degree.
Kanar et al. 2020 [13]	DME IVA (*n* = 28 eyes); IVA + SDM (*n* = 28 eyes)	Prospective RCT; comparison of BCVA, CRT, and number of injections required in both groups at 12 months; SDM applied after at least three loading doses of IVA and until CRT decreased below 450 µm.	IVA group experienced significant BCVA improvement from 0.38 ± 0.1 logMAR to 0.20 ± 0.1 logMAR and CRT reduction from 451.28 ± 44.85 µm to 328.8 ± 49.69 µm, while the combined group experienced significant BCVA improvement from 0.40 ± 0.09 logMAR to 0.17 ± 0.06 logMAR and CRT reduction from 466.07 ± 71.79 µm to 312.0 ± 39.29 µm—thus, no statistically significant differences in BCVA and CRT changes existed between the groups; the number of injections in the combined group was significantly smaller than in the monotherapy group at 3.21 ± 0.41 vs. 5.39 ± 1.54.
**BRVO**			
Terashima et al. 2019 [14]	ME secondary to BRVO IVR group (*n* = 24 eyes); IVR + SDM group (*n* = 22 eyes)	Retrospective; evaluation of BCVA, CRT, and number of injections in both groups at six months; SDM performed one month after initial IVR; IVR applied in PRN fashion after the first initial injection in both groups.	BCVA and CRT improved significantly in both groups without significant differences; combined group required statistically fewer injections than the IVR monotherapy group (1.9 ± 0.8 vs. 2.3 ± 0.9) by three months.

SDM, subthreshold diode micropulsation; IVR, intravitreal ranibizumab; IVA, intravitreal aflibercept; IVT, intravitreal triamcinolone; BCVA, best-corrected visual acuity; CRT, central retinal thickness; ME, macular edema; BRVO, branch retinal vein occlusion; DME, diabetic macular edema; CS, contrast sensitivity; RCT, randomized clinical trial; PRN, pro re nata; VEGF, vascular endothelial growth factor.

**Table 2:** Studies that assessed the combination of SDM and intravitreal treatment without results for intravitreal therapy alone.

Author/Year of Publication	Material	Study Design	Results
**BRVO**			
Parodi et al. 2008 [15]	ME secondary to BRVO SDM (*n* = 13 eyes) (810 nm) SDM + IVT (*n* = 11 eyes)	Prospective RCT; comparison of BCVA between the groups at 12 months.	Gain of at least 10 ETDRS letters in 91% of eyes in the SDM + IVT group and in 62% of eyes in the SDM-alone group; mean number of lines gained: 3.4 in the SDM + IVT group and 1.3 in the SDM-alone groups (the difference between the groups was significant).
**DME**			
Luttrull et al. 2012 [16]	DME SDM (*n* = 38 eyes); SDM + anti-VEGF or IVT (*n* = 24 eyes); SDM-alone group had significantly smaller CRT at baseline	Retrospective; evaluation of BCVA and CRT after treatment (median follow up 12 months); SDM followed intravitreal therapy.	Significant reduction in CRT in 71% of the SDM-alone group and 89.5% of the combination group (with no statistical difference between the groups); BCVA stable in both groups, but without significant improvement.
Elhamid 2017 [17]	DME resistant to anti-VEGF therapy Ozurdex* plus SDM (*n* = 20 eyes)	Case series; evaluation of BCVA and CRT at 12 months; SDM performed at one month after injection of Ozurdex; possible reinjection at six months.	BCVA was significantly improved from 0.45 ± 0.14 to 0.59 ± 0.14 Snellen, while CRT was significantly reduced from 420.7 ± 38.74 μm to 285.2 ± 14.99 μm; reinjection was necessary in eight eyes; cataract was present in six of 14 phakic eyes.
Inagaki et al. 2019 [18]	DME SDM + anti-VEGF (*n* = 34 eyes, including 27 IVR and 7 IVA)	Retrospective; evaluation of BCVA, CRT, and the number of injections at 12 months; loading dose of anti-VEGF until ME disappearance, then SDM within a month and, after that, anti-VEGF was delivered in PRN fashion.	BCVA: significant improvement from 0.52 ± 0.34 logMAR to 0.41 ± 0.34 logMAR at 12 months; stable reduction of CRT through 12 months from 491.1 ± 133.9 μm to 354.8 ± 120.4 μm; mean number of injections: 3.6 ± 2.1 during one year.

SDM, subthreshold diode micropulsation; IVR, intravitreal ranibizumab; IVA, intravitreal aflibercept; IVT, intravitreal triamcinolone; BCVA, best-corrected visual acuity; CRT, central retinal thickness; ME, macular edema; BRVO, branch retinal vein occlusion; DME, diabetic macular edema; CS, contrast sensitivity; ETDRS—Early Treatment Diabetic Retinopathy Study, RCT, randomized clinical trial; PRN, pro re nata. *, Manufactured by Allergan, Dublin, Ireland.

## Data Availability

No new data were created or analyzed in this study. Data sharing is not applicable to this article.
